# Metal–Phenolic Nanomedicines Targeting Fatty Acid Metabolic Reprogramming to Overcome Immunosuppression in Radiometabolic Cancer Therapy

**DOI:** 10.1021/acsami.4c21028

**Published:** 2025-01-28

**Authors:** Guohao Wang, Dongmei Wang, Lu Xia, Jiabian Lian, Qing Zhang, Dongyan Shen, Zhanxiang Wang, Yunlu Dai

**Affiliations:** ‡ Xiamen Cell Therapy Research Center, The First Affiliated Hospital of Xiamen University, School of Medicine, Xiamen University, Xiamen 361003, China; § Department of Public Health and Medical Technology, Xiamen Medical College, Xiamen 361023, China; ⊥ Center for Precision Medicine, The First Affiliated Hospital, School of Medicine, Xiamen University, Xiamen361000, China; ∥ Department of Laboratory Medicine, The First Affiliated Hospital, School of Medicine, Xiamen University, Xiamen361000, China; ¶ Department of Cardiology, The First Affiliated Hospital of Xiamen University, School of Medicine, Xiamen University, Xiamen 361003, China; # Department of Neurosurgery and Department of Neuroscience, Fujian Key Laboratory of Brain Tumors Diagnosis and Precision Treatment, Xiamen Key Laboratory of Brain Center, the First Affiliated Hospital of Xiamen University, School of Medicine, Xiamen University, Xiamen 361003, China; ∇ Cancer Centre and Institute of Translational Medicine, Faculty of Health Sciences, University of Macau, Macau SAR 999078, China; ○ MoE Frontiers Science Center for Precision Oncology, University of Macau, Macau SAR 999078, China

**Keywords:** fatty acid, cancer radiotherapy, radiosensitizer, metal−phenolic networks, immunotherapy

## Abstract

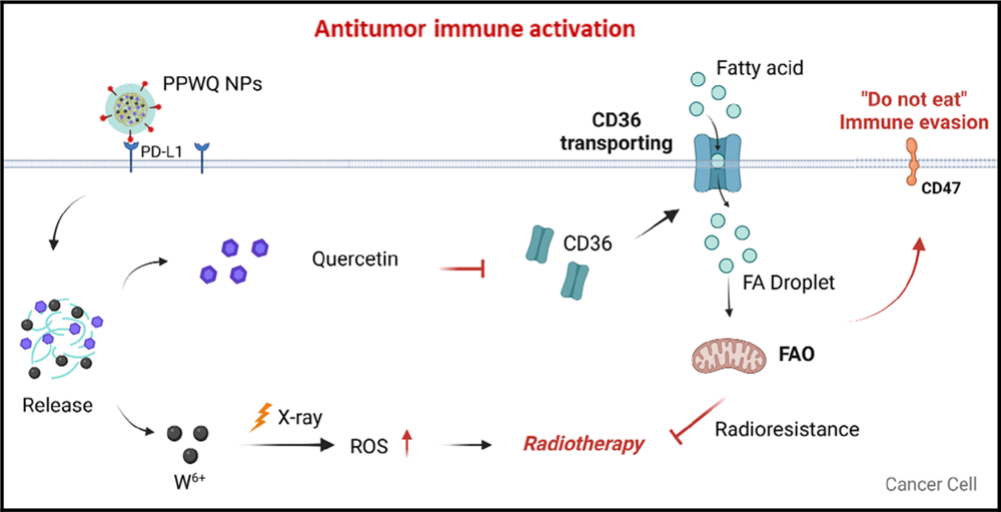

Radiation therapy (RT) is a prevalent cancer treatment; however, its therapeutic outcomes are frequently impeded by tumor radioresistance, largely attributed to metabolic reprogramming characterized by increased fatty acid uptake and oxidation. To overcome this limitation, we developed polyphenol–metal coordination polymer (PPWQ), a novel nanoradiotherapy sensitizer specifically designed to regulate fatty acid metabolism and improve RT efficacy. These nanoparticles (NPs) utilize a metal–phenolic network (MPN) to integrate tungsten ions (W^6+^), quercetin (QR), and a PD-L1-blocking peptide within a PEG–polyphenol scaffold. When exposed to X-rays, PPWQ induces reactive oxygen species (ROS) to cause DNA damage, while QR inhibits CD36 expression, effectively curbing fatty acid uptake and mitigating immune evasion. In a 4T1 tumor-bearing mouse model, PPWQ demonstrated significant enhancement of RT by facilitating dendritic cell activation, boosting memory cytotoxic T lymphocytes, and skewing macrophages toward a pro-immune phenotype. These results underscore the potential of PPWQ to target metabolic vulnerabilities and advance the integration of immunotherapy with radiotherapy.

## Introduction

Radiation therapy (RT) is a cornerstone of clinical cancer treatment; however, its therapeutic potential is often constrained by radiation resistance.^1−3^ Emerging evidence links this resistance to metabolic reprogramming in tumor cells, particularly the enhanced uptake of exogenous fatty acids, which supports their adaptation to radiation-induced stress.^4^ This increased fatty acid uptake fuels fatty acid oxidation (FAO), fulfilling the energy demands under oxidative stress and supplying metabolic intermediates crucial for DNA damage repair, thereby promoting tumor survival and resistance.^5^ Furthermore, elevated intracellular fatty acids upregulate CD47 expression on tumor cell surfaces.^6^ Acting as a “do-not-eat-me” signal, CD47 impedes macrophage phagocytosis, allowing tumor cells to evade immune clearance.^7,8^ Tumor cells also suppress cytotoxic T lymphocyte (CTL) activity by depleting essential nutrients like glucose and glutamine in the tumor microenvironment (TME), weakening CTL-mediated cytotoxicity.^9,10^ These processes not only bolster tumor radioresistance but also foster an immunosuppressive microenvironment enriched with regulatory T cells and myeloid-derived suppressor cells, exacerbating immune evasion. The dual role of fatty acid metabolism in DNA repair and immune suppression underscores the intricate interplay between metabolic reprogramming and the TME, offering novel insights into radiation resistance. Targeting fatty acid metabolism, such as inhibiting fatty acid uptake or disrupting CD47 signaling, holds promise as a strategy to overcome resistance and improve RT efficacy.

CD36, a key fatty acid transporter, plays a central role in mediating fatty acid uptake by binding free fatty acids with high affinity and facilitating their transmembrane transport.^11,12^ It enhances fatty acid binding efficiency by localizing to lipid rafts and employs a flipping mechanism to import fatty acids into the cell. In conjunction with fatty acid-binding proteins and acyl-CoA synthetases, CD36 activates fatty acids into acyl-CoA for mitochondrial oxidation, generating ATP to meet energy demands.^13−15^ Radiation-induced oxidative stress upregulates CD36, further supporting tumor cell survival by providing energy for DNA repair.^16,17^ Additionally, metabolic byproducts of FAO regulate cellular signaling pathways, reinforcing resistance to radiation.^18−20^ This radiation-driven metabolic reprogramming is particularly pronounced in certain tumors, highlighting CD36-mediated fatty acid transport as a promising therapeutic target.

**Scheme 1:**
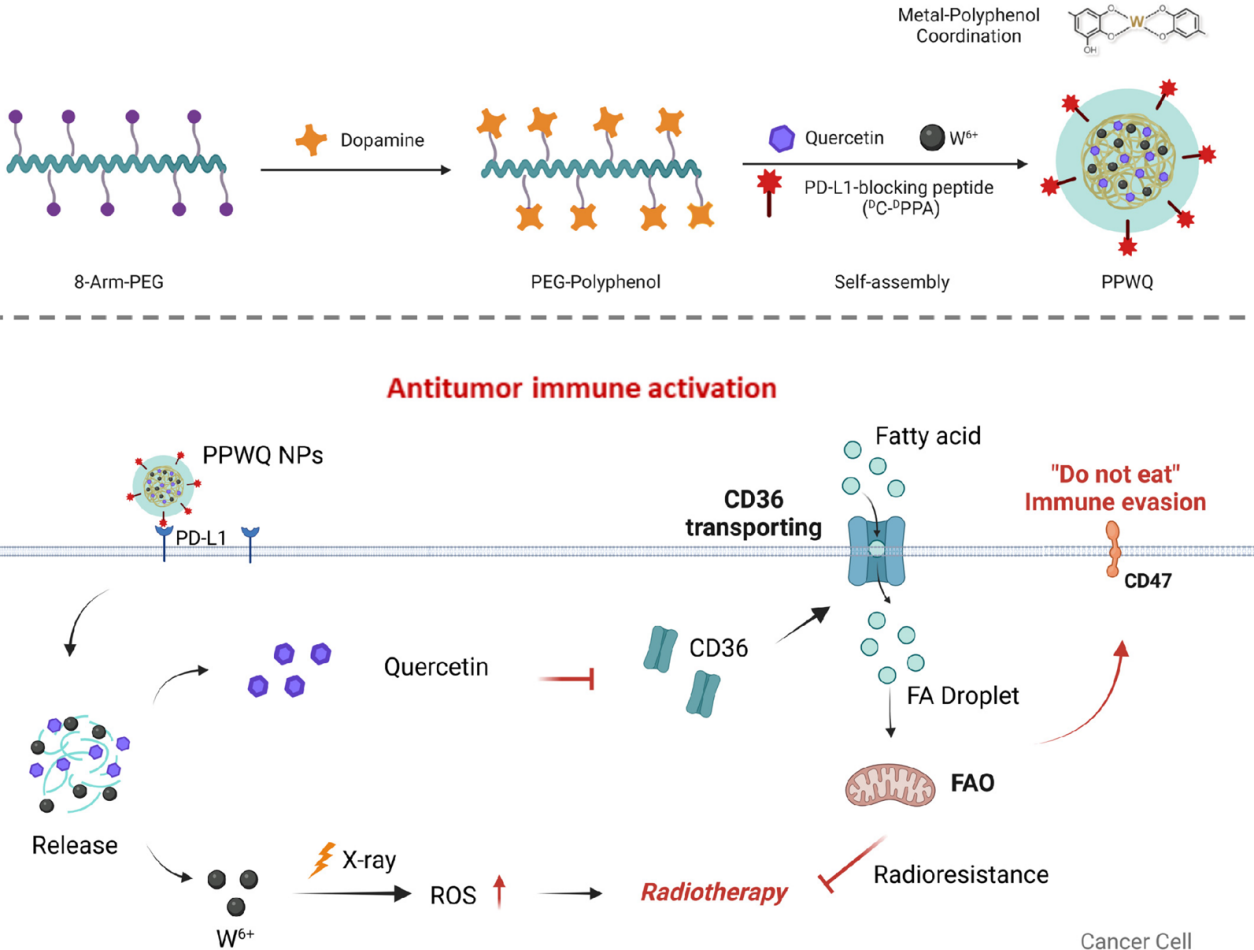
PPWQ Employing a PD-L1-Blocking Peptide to Selectively Target Breast Cancer Cells with Elevated PD-L1 Expression, Enabling Precise Accumulation in Tumor Tissues Intracellularly released W^6+^ act as radiosensitizers, amplifying ROS generation and enhancing DNA damage caused by radiotherapy. Simultaneously, QR suppresses CD36 expression, decreasing fatty acid uptake by tumor cells within the TME and mitigating radiotherapy resistance. Furthermore, QR downregulates CD47 expression on tumor cell surfaces, reducing immune evasion and promoting immune-mediated clearance. This dual-action mechanism synergistically boosts the efficacy of radiotherapy while activating an anti-tumor immune response.

Nanomedicines offer the advantage of tumor-specific accumulation with minimal off-target effects.^21^ Among these, metal–phenolic networks (MPNs), which are synthesized by the interaction of polyphenols with metal ions, hold significant promise for applications in targeted drug delivery, bioimaging, and cancer treatment.^22^ In this study, we developed an advanced nanoradiotherapy sensitizer, polyphenol–metal coordination polymer (PPWQ), designed to modulate fatty acid metabolism during radiotherapy. PPWQ incorporates a PD-L1-blocking peptide (^D^C-^D^PPA, ^D^C-^D^N^D^Y^D^S^D^K^D^P^D^T^D^D^D^R^D^Q^D^Y^D^H^D^F) for enhanced tumor targeting and immune modulation, utilizing MPNs to coordinate tungsten ions (W^6+^) with PEG–polyphenol while embedding quercetin (QR) in its hydrophobic core (Scheme 1). These nanoparticles (NPs) effectively target tumors with high PD-L1 expression. Upon X-ray irradiation, the W^6+^ ions generate reactive oxygen species (ROS), inducing DNA strand breaks. Simultaneously, QR suppresses CD36 expression epigenetically, reducing fatty acid uptake, inhibiting tumor proliferation, and downregulating CD47 expression. In a 4T1 tumor-bearing mouse model, PPWQ significantly enhanced radiotherapy by promoting dendritic cell (DC) maturation, recruiting memory phenotype CTLs, and reprogramming tumor-associated macrophages (TAMs) toward an immune-supportive phenotype. By integrating fatty acid metabolic modulation with radiotherapy, this approach introduces a novel pathway to advance immunoradiotherapy.

## Results and Discussion

### Synthesis and Characterization of PPWQ NPs

MPNs, formed through the coordination of metal ions with phenolic molecules, have emerged as versatile and promising nanoplatforms in the field of biomedicine.^23^ Their wide-ranging functionalities and applications stem from the extensive variety of available metal ions and phenolic compounds. QR, a flavonoid compound containing polyphenolic groups, has been shown to effectively inhibit cancer progression by modulating cell proliferation signaling pathways.^24^

**Figure 1:**
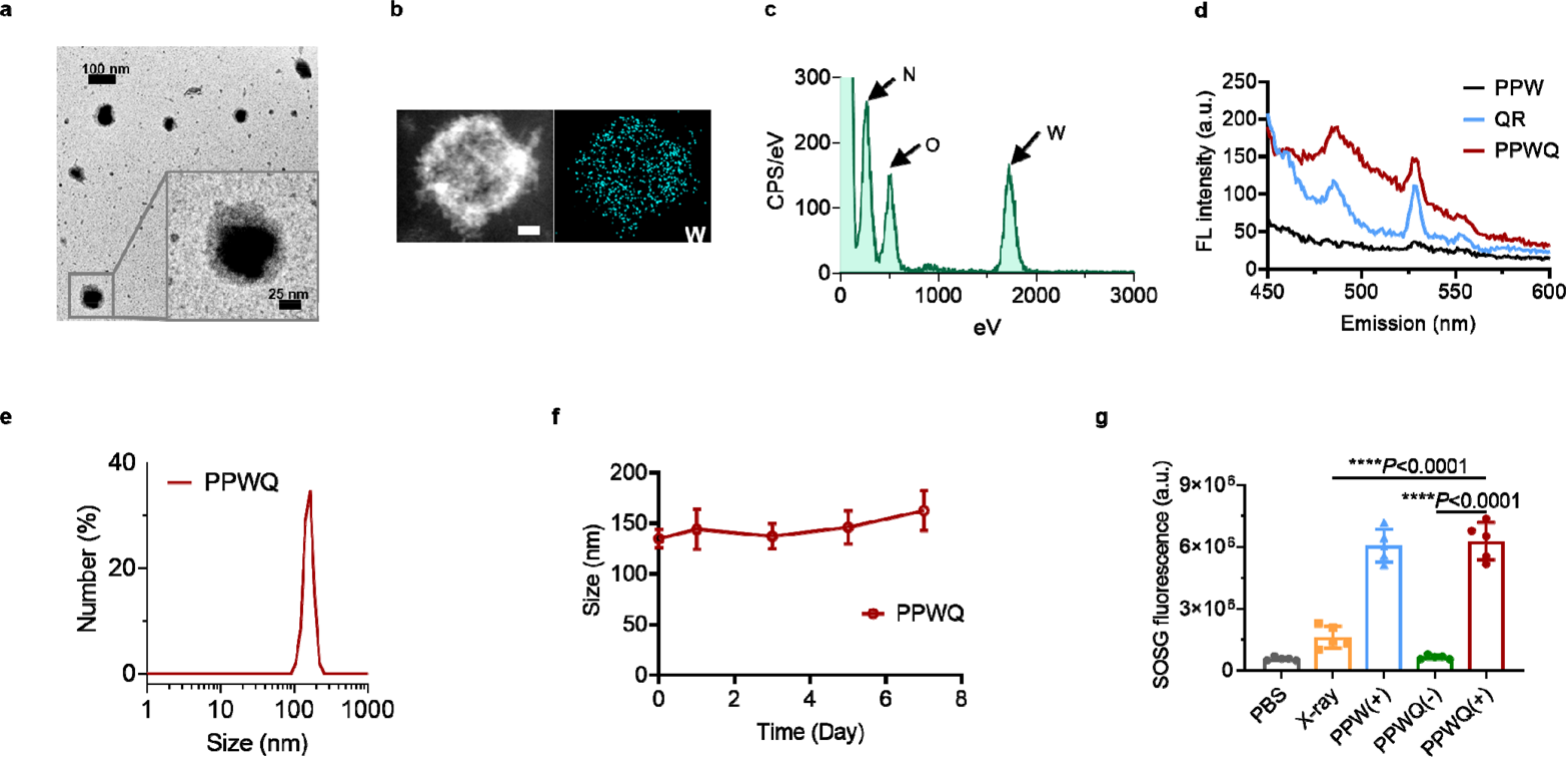
Characterization of PPWQ NPs. (a) The morphology of PPWQ NPs was characterized using TEM, with images captured at scale bars of 100 nm and 25 nm. (b and c) Elemental mapping and EDS provided confirmation of the NP composition, with a finer scale bar of 20 nm. (d) Fluorescence spectroscopy characterized PPW, QR and PPWQ NPs. (e) Dynamic light scattering (DLS) analysis measured the particle size, yielding an average diameter of 143 ± 10.7 nm. (f) The stability of PPWQ NPs was monitored over a 1-week period. (g) Singlet oxygen (^1^O_2_) generation was quantified through SOSG fluorescence analysis, with the results presented as mean ± SD (*n* = 3).

However, the poor water solubility of QR presents a major challenge to its effective application in cancer therapy. In addition, RT is known to induce the upregulation of PD-L1 expression in tumor cells, which compromises the activity of tumor-infiltrating immune cells.^25,26^ In this project, we developed PPWQ NPs by coordinating ^D^C-^D^PPA (P), W^6+^ ions, and QR with PEG–polyphenol through metal-phenolic interactions. The synthesis of the PEG–polyphenol derivative was based on methods established in our prior research. Subsequently, PEG–polyphenol, P, W^6+^ ions, and QR were sequentially combined, resulting in the formation of MPN-based PPWQ NPs through a streamlined one-pot self-assembly process. Transmission electron microscopy (TEM) revealed that PPWQ NPs exhibit a uniform spherical morphology with a well-defined core–shell structure (Figure 1a). Elemental mapping and energy-dispersive X-ray spectroscopy (EDS) verified the tungsten incorporation and overall NP morphology (Figure 1b,c). To validate the encapsulation of QR in PPWQ NPs, we employed both fluorescence and UV–vis spectroscopy. Fluorescence spectra revealed a similar emission peak at 460 nm, consistent with QR’s fluorescence, supporting its encapsulation (Figure 1d). UV–vis analysis showed a distinct absorbance peak at 370 nm in PPWQ, corresponding to QR’s characteristic peak, further confirming its incorporation into the NPs (Figure S1). Together with the ζ-potential data, which increased from 6.22 mV (PEG) to 12.93 mV (PPWQ), these results provide compelling evidence that W and QR have been successfully encapsulated within the PPWQ formulation (Figure S2). Encapsulation efficiency for W^6+^ ions was quantified using inductively coupled plasma mass spectrometry (ICP-MS). As summarized in Tables S1, W^6+^ ions were evenly encapsulated with a loading content of 11.99% and an efficiency exceeding 86%, based on a PP/WCl_6_/QR weight ratio of 30:5:1. The PPWQ NPs demonstrated excellent stability, retaining an average size of 143 ± 10.7 nm over 7 days (Figure 1e). PPWQ NPs showed negligible size variations after incubation in saline containing 10% fetal bovine serum for 7 days (Figures 1f and S3). To evaluate the ability of the radiosensitizer W^6+^ to generate singlet oxygen (^1^O_2_), we performed singlet oxygen sensor green (SOSG) assays. As shown in Figure 1g, X-ray irradiation significantly enhanced the ^1^O_2_ levels in both PPW (PEG–polyphenol loaded with P and W^6+^) and PPWQ NPs, demonstrating their potential as effective radiosensitizers.

### PPWQ NPs Impeding the Uptake of Fatty Acids by 4T1 Cells through Downregulation of the CD36 Expression

**Figure 2:**
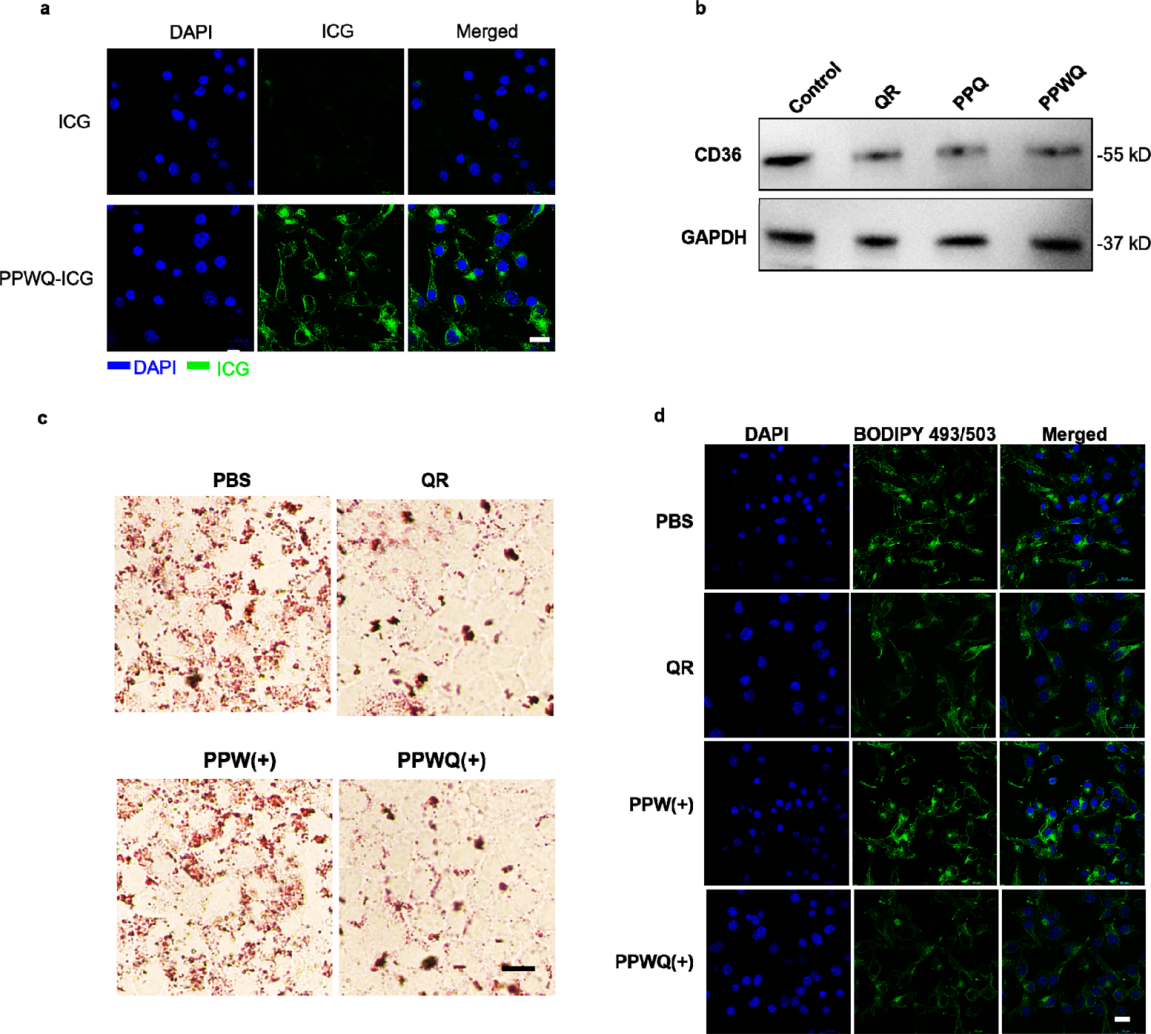
PPWQ inhibiting fatty acid uptake by 4T1 cells via CD36 downregulation. (a) Confocal microscopy illustrated the intracellular distribution of ICG-labeled PPWQ NPs, with PEG conjugated to ICG visualized in green and cell nuclei counterstained with DAPI in blue. The images were captured using a scale bar of 20 μm. (b) Western blot analysis revealed CD36 expression levels in 4T1 cells following treatment with different agents, using GAPDH as an internal loading control. (c) Lipid accumulation in 4T1 cells was demonstrated by oil red O staining, with the scale bar set to 20 μm. (d) Lipid droplets within 4T1 cells were detected using BODIPY 493/503 staining, with a scale bar of 20 μm.

QR inhibits CD36 expression through transcriptional regulation via suppression of key transcription factors like PPARγ and NF-κB, as well as epigenetic modulation, including DNA methylation and histone modification.^27^ First, the successful endocytic uptake of PPWQ NPs was confirmed by detecting fluorescence in the cytoplasm following an 8-h incubation with ICG-labeled PPWQ NPs. ICG labeling was achieved by conjugating the free NHS groups of PEG–polyphenol. As shown in Figure 2a, ^D^C-^D^PPA-based PPWQ NPs specifically targeted 4T1 cancer cells exhibiting PD-L1 overexpression, as evidenced by the significant reduction in uptake when pretreated with anti-PD-L1 antibodies (Figure S4). To further explore the regulatory effect of QR on CD36 expression, we observed that QR significantly suppressed CD36 levels in tumor cells (Figures 2b and S5). Co-loading QR with PPWQ amplified this inhibitory effect, demonstrating a pronounced advantage in reducing CD36 expression compared to QR alone. Interestingly, radiation sensitization with PPW NPs combined with X-ray irradiation led to a significant increase in CD36 expression levels compared to the blank control group, as shown in Figures S6 and S7.

Next, we investigated the impact of QR on fatty acid uptake in 4T1 tumor cells. As shown in Figure 2c, QR-treated tumor cells exhibited significantly reduced intracellular fatty acid levels compared to the control group. Conversely, RT [PPW(+)] resulted in a significant increase in fatty acid uptake, likely due to the heightened demand for energy and metabolic intermediates required for RT-induced DNA damage repair. However, treatment with PPWQ-sensitized RT significantly inhibited fatty acid uptake, effectively impairing the tumor cells’ self-repair mechanisms. To confirm these changes, fluorescent probe labeling and confocal laser scanning microscopy were used to monitor fatty acid distribution and uptake. The results, consistent with earlier observations, showed that both QR and PPWQ treatments effectively reduced fatty acid uptake in tumor cells (Figure 2d). Collectively, these findings suggest that QR inhibits CD36 expression, reducing the uptake of exogenous fatty acids and disrupting metabolic reprogramming, thereby impairing the self-repair mechanisms of tumor cells following RT.

### Reprogrammed Fatty Acid-Rich Microenvironment by PPWQ Regulating DC and Macrophage Cell Function

**Figure 3:**
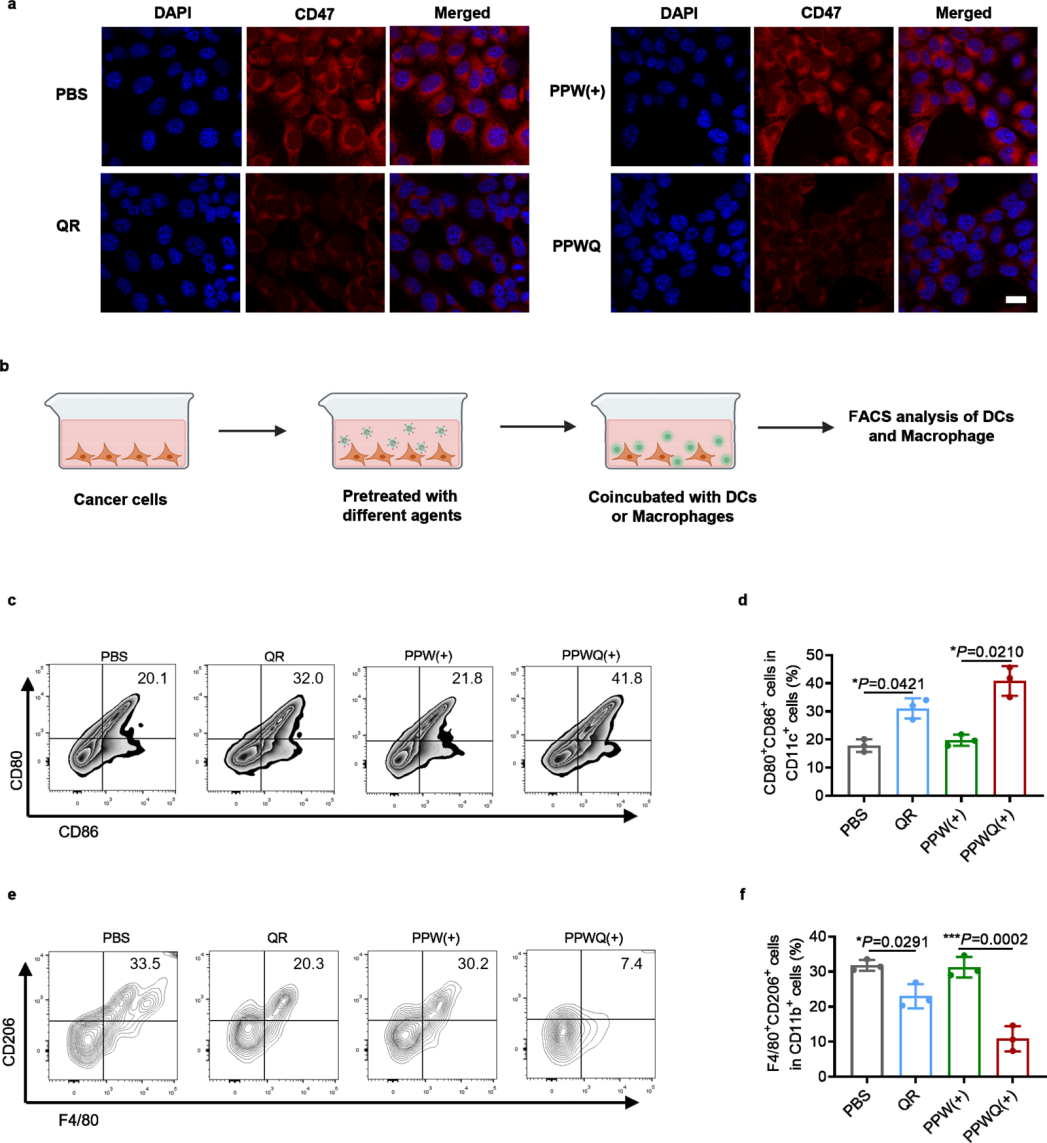
Modulation of DC and macrophage function by PPWQ. (a) Confocal microscopy demonstrated CD47 expression on 4T1 cells following 72 h of incubation, with a scale bar of 20 μm. (b) A schematic illustration depicted the coculture system involving tumor cells, DCs, and macrophages. Flow cytometry analysis (c) and corresponding quantification (d) showed the proportions of mature DCs (CD11c^+^CD80^+^CD86^+^) after coculture with 4T1 cells subjected to various treatments. Flow cytometry plots (e) and quantitative analysis (f) revealed the prevalence of M2-like macrophages (CD206^+^) gated on CD45^+^CD11b^+^F4/80^+^ cells. The data, representing mean ± SD from three biological replicates (*n* = 3), were statistically evaluated using one-way ANOVA followed by Tukey’s posthoc tests.

Under normal physiological conditions, the accumulation of fatty acids in tumor cells significantly promotes the expression of CD47, a “do-not-eat-me” signal that enables tumor cells to evade recognition and clearance by macrophages. To investigate the effects of QR and its PPWQ on CD47 expression levels in tumor cells, we conducted immunofluorescence analysis (Figure 3a). The results revealed that both untreated tumor cells and those exposed to X-rays expressed high levels of CD47 on their cell membrane surfaces. However, treatment with QR or PPWQ markedly reduced CD47 expression, suggesting that QR and its polymeric form may inhibit fatty acid accumulation and consequently reduce the immune evasion capacity of tumor cells.

DCs rely on exogenous fatty acids for maturation, proliferation, and effector functions. Next, we explored whether disrupting fatty acid uptake in tumor cells could alter their metabolic competition with neighboring DCs. Tumor cells pretreated with PBS, QR, PPW(+) or PPWQ(+) to regulate CD36 expression were collected and cocultured with DCs to track the competition for fatty acid uptake (Figure 3b). Flow cytometry analysis showed a significant increase in the proportion of mature DCs in QR- and PPWQ-treated groups compared to the control. Furthermore, the PPWQ group exhibited higher DC maturation levels than the group treated with radiation sensitization alone, demonstrating PPWQ’s superiority in enhancing DC maturation (Figures 3c,d and S8). Macrophages also utilize fatty acids to support polarization toward the M1 phenotype, which is critical for antitumor activity. Tumor cells pretreated with QR or PPWQ to regulate CD36 expression were cocultured with macrophages. In these conditions, fatty acids in the culture medium were preserved as tumor cells consumed fewer fatty acids, enabling macrophages to polarize more effectively toward the M1 phenotype. Flow cytometry further validated this observation, showing a significantly higher proportion of M2 macrophages in the control and radiation sensitization groups compared to the PPWQ group. In contrast, the PPWQ-treated group exhibited a marked increase in the proportion of M1 macrophages (Figures 3e,f and S9). These findings indicate that reducing fatty acid consumption by inhibiting CD36 expression in tumor cells provides more fatty acid resources for DCs and macrophages, thereby enhancing the functional capacity of immune cells. In conclusion, QR and PPWQ regulate the expression of CD36 and CD47 in tumor cells, not only inhibiting immune evasion but also improving metabolic competition to promote DC maturation and macrophage M1 polarization.

### In Vitro Radiosensitization Effect of PPWQ NPs

**Figure 4:**
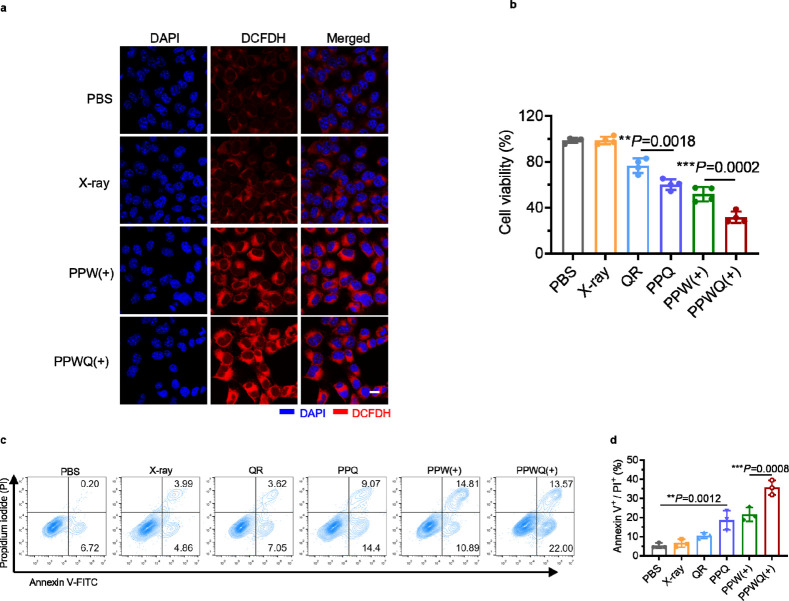
PPWQ amplification of the tumoricidal efficacy of radiotherapy. (a) Representative CLSM images of 4T1 cells were stained with the ROS probe 2′,7′-dichlorodihydrofluorescein diacetate (DCFH-DA) after different treatments [PBS, X-ray, PPW(+), or PPWQ(+)] over a 3-day period (*n* = 3). The symbols (+) and (−) denote treatments with or without 6 Gy X-ray irradiation, respectively. Scale bar: 20 μm. (b) The cytotoxic effects of PPWQ NPs on 4T1 cells under X-ray irradiation were evaluated using MTT assay. Flow cytometry plots (c) and their quantification (d) illustrate the proportions of PI- and Annexin V-FITC-stained 4T1 cells under the specified treatment conditions.

RT destroys cancer cells by generating high levels of ROS, which induce DNA strand breaks. However, cancer cells can counteract this DNA damage by upregulating the expression of the fatty acid transporter CD36, thereby increasing fatty acid uptake to protect themselves from apoptosis.^17,28^ As shown in Figure 4a, X-ray irradiation activated strong ROS fluorescence signals in 4T1 cancer cells treated with both PPW and PPWQ NPs [labeled as PPW(+) and PPWQ(+), respectively]. Notably, the therapeutic efficacy of the PPWQ(+) group in suppressing cell viability was significantly superior to that of the PPW(+) group (Figure 4b–d). Previous analyses of CD36 levels and fatty acid accumulation revealed contrasting responses between the two treatment groups. In the PPW(+) group, CD36 expression was significantly upregulated, leading to increased fatty acid uptake. In contrast, the PPWQ(+) group, which incorporated QR, successfully suppressed CD36 expression and markedly reduced fatty acid accumulation. Specifically, the PPW(+) treatment may have facilitated cancer cell resistance to ROS-induced DNA damage by enhancing CD36 expression and supplying fatty acids for survival. In the PPWQ(+) group, QR effectively inhibited CD36 upregulation, blocking fatty acid uptake. This, in turn, inhibited tumor cells from utilizing FAO to produce the energy and metabolic intermediates required for DNA repair. Thus, the high atomic number W^6+^ in PPWQ acted as X-ray sensitizers, significantly enhancing ROS generation and exacerbating DNA damage. Additionally, through the combined action of QR, PPWQ inhibited the CD36-dependent fatty acid uptake and DNA repair pathways, ultimately suppressing tumor cell growth and proliferation.

### Amplifiable Effects of PPWQ in Downregulating Fatty Acid Uptake and Initiating the Immune Response in Vivo

To evaluate the biodistribution of PPWQ in vivo, an orthotopic 4T1 breast cancer mouse model was established by injecting 1 million 4T1 cells into the fourth mammary fat pad of mice. When tumor volumes reached approximately 100 mm^3^, the study commenced with intravenous injections of ICG-labeling PPWQ. As shown in Figure S10, PPWQ began accumulating in tumor tissues 4 h postinjection (highlighted by red circles) and reached a stable level by 24 h. Fluorescence signals in major organs, including tumors, gradually diminished over the subsequent 2 days, indicating efficient metabolism and clearance. At the end of the study, fluorescence quantification demonstrated a markedly higher signal intensity in tumor tissues compared to other organs, such as the heart, liver, lung, kidney, and spleen, 48 h postinjection (Figure S11). These results demonstrate the tumor-targeted delivery capability of PPWQ, achieving high tumor enrichment while minimizing accumulation in nontarget tissues. Furthermore, the biodegradable properties of PPWQ reduce potential toxicity to normal tissues, confirming its biocompatibility.

**Figure 5:**
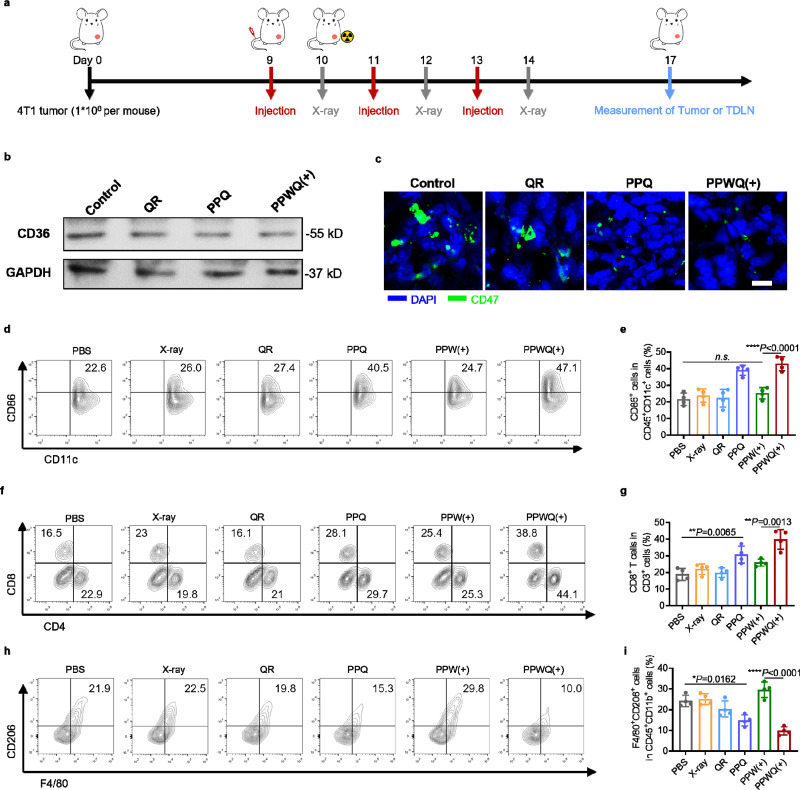
PPWQ-enhanced CD36 inhibition and immune activation in vivo. (a) Schematic of the experimental timeline for parts b–i. Tumor-bearing mice were treated with PBS, X-ray, QR, PPQ, PPW(+), or PPWQ(+), where (+) and (−) indicate the presence or absence of 6 Gy X-ray irradiation. Tumors were harvested on day 17 for analysis. (b) Western blot analysis of CD36 expression in tumor tissues was performed, with GAPDH as the loading control. (c) Confocal images of tumor sections stained for CD47 (green) and nuclei (blue) are provided. Scale bar: 10 μm. Flow cytometry analysis (d) and quantification (e) of CD86^+^ activated DCs gated on CD45^+^CD11c^+^ cells in TDLNs are provided. Flow cytometry plots (f) and quantification (g) of CD8^+^ T cells gated on CD3^+^ T cells in tumor tissues are provided. Flow cytometry plots (h) and quantification (i) of M2-like macrophages (CD206^+^) gated on CD45^+^CD11b^+^F4/80^+^ cells are provided.

To assess the effects of PPWQ on intratumoral CD36 expression and immune responses, mice bearing orthotopic 4T1 breast tumors were randomly assigned to six groups and received tail vein injections according to the treatment protocol (Figure 5a). For groups subjected to X-ray irradiation, tumors were exposed to a 6 Gy dose 24 h after drug injection. Treatments were administered three times, and tumor tissues and tumor-draining lymph nodes (TDLNs) were harvested on the third day following the final treatment for analysis. Western blot analysis revealed reduced CD36 expression levels in tumors treated with PPQ or PPWQ (Figures 5b and S12), consistent with prior in vitro findings. The downregulation of CD36 likely inhibits fatty acid uptake in tumor cells, thereby reducing fatty acid-driven CD47 expression. Immunofluorescence analysis showed markedly weaker CD47 signals in tumor sections from the PPQ and PPWQ groups in comparison to the PBS group (Figure 5c), suggesting enhanced macrophage-mediated antitumor activity and immune clearance.

Fatty acid content in the TME is critical for antigen presentation and immune regulation.^29^ To explore treatment-induced immune responses, the levels of stimulatory molecules, such as CD86, on DCs in TDLNs were evaluated (Figure 5d,e). Neither X-ray irradiation alone nor radiation sensitizer treatments significantly increased the proportion of mature DCs (CD86^+^CD11c^+^) compared to the PBS group. However, combining QR and PPWQ with X-ray irradiation significantly enhanced DC maturation, enabling effective phagocytosis of cancer antigens and cross-presentation to activate CD8^+^ T cells. Following treatment, the proportion of CD8^+^CD3^+^ T cells in tumors was 2.11-fold and 1.54-fold higher in the PPWQ+X-ray group compared to the PBS and PPW(+) groups, respectively (Figures 5f,g, S13, and S14).

We also examined macrophage polarization in tumor tissues across treatment groups. The PPW(+) group displayed a higher proportion of M2 macrophages (Figure 5h,i), indicating that RT alone may foster an immunosuppressive microenvironment. In contrast, PPQ treatment markedly decreased the proportion of M2 macrophages, promoting polarization toward the M1 phenotype. The combined application of QR and radiation sensitizers reversed radiation-induced immunosuppression, transforming the TME into an immune-supportive state. In conclusion, the combination of QR and radiation sensitizers downregulated CD36 and CD47 expression in tumor tissues, improved fatty acid metabolism in the TME, enhanced the immune activity of DCs and CD8^+^ T cells, and promoted M1 macrophage polarization, thereby overcoming radiation-induced immunosuppression.

### Tumor Growth Suppression and Immune Response after Treatment

**Figure 6:**
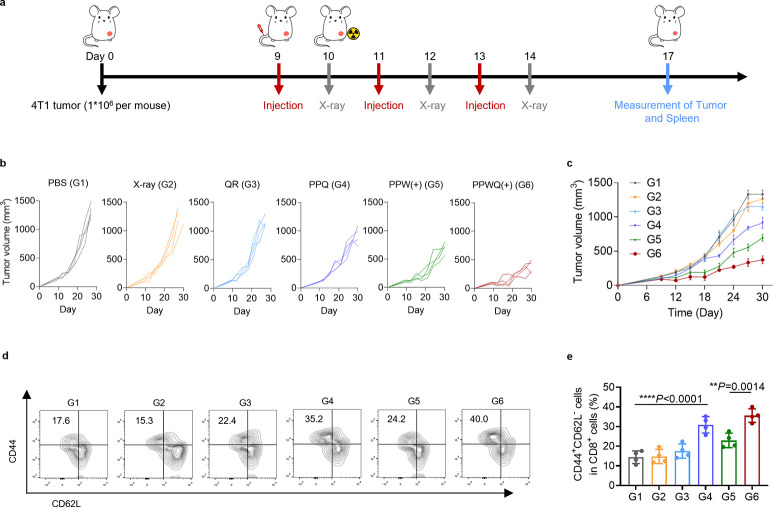
PPWQ combined with radiotherapy inducing effective therapeutic effects. (a) A schematic diagram illustrates the therapeutic schedule corresponding to panels b–e. Tumor growth in treated mice was assessed through individual tumor growth curves (b) and the average tumor growth rates (c) across treatment groups. Flow cytometry analysis was conducted to evaluate memory T cell populations (CD3^+^CD8^+^CD44^+^CD62L^–^) in the spleens of treated mice, with the results presented as flow cytometry plots (d) and quantification (e).

Next, we evaluated the antitumor efficacy of PPWQ in a mouse orthotopic 4T1 breast tumor model (Figure 6a). Tumors were established by orthotopically implanting 4T1 cells into the fourth right mammary fat pad of mice, serving as targets for localized X-ray irradiation. Mice received intravenous injections of PBS, QR, PPQ, PPW, or PPWQ on days 9, 11, and 13. A total of 24 h postinjection, tumors were irradiated with 6 Gy of X-rays, and tumor growth was monitored for up to 30 days. Tumor growth in mice treated with X-ray irradiation alone showed no significant delay compared to the PBS group (Figure 6b,c). The PPQ group demonstrated moderate tumor growth suppression, likely due to inhibited fatty acid uptake. However, when combined with X-ray irradiation, PPWQ treatment significantly reduced tumor volume. This effect was attributed to the synergistic impact of metabolic reprogramming within the TME and boosted antitumor immune responses, substantially improving the efficacy of radiotherapy.

Further analysis revealed that PPWQ(+) treatment not only eradicated tumor cells but also enhanced macrophage-mediated antitumor activity and DC activation, leading to robust stimulation of immune memory. As shown in Figures 6d,e and S15, the proportion of memory CD8^+^ T cells (CD44^+^CD62L^–^) in the PPWQ(+) group was 2.47 times higher than that in the control group. Moreover, PPWQ(+) demonstrated superior enhancement of antitumor immune memory compared to the PPW(+) group. While these results are promising, potential toxicity and the uncertain nanobio interactions of nanotherapeutics remain challenges for clinical application. To address these concerns, multiple biomedical engineering strategies were employed to optimize the biosafety of PPWQ. The nanocarrier was constructed using PEG–polyphenol, a material known for its excellent biocompatibility. The polyphenol component, derived from natural plants, provided superior biodegradability and biosafety. Additionally, the robust coordination between polyphenols and metal ions provided outstanding physiological stability, while PD-L1 blocking peptide modification enhanced tumor targeting and immunogenicity. Throughout the experiment, the stable body weight and normal growth of mice corroborated the treatment’s biosafety and biocompatibility. These results underscore the clinical potential of PPWQ as a safe and effective nanotherapeutic (Figure S16).

## Conclusions

This study presents a novel approach combining metabolic reprogramming with radiotherapy, demonstrating that QR-mediated inhibition of CD36 enhances tumor radiosensitivity and challenges traditional paradigms by targeting both metabolic and immune resistance mechanisms. Our findings demonstrate that PPWQ enhances the efficacy of RT through a dual mechanism/W^6+^ generate ROS, inducing extensive DNA damage in tumor cells, while QR suppresses CD36 expression, thereby reducing fatty acid uptake and mitigating CD47-mediated immune evasion. This strategy disrupts tumor metabolic pathways essential for DNA repair and simultaneously reprograms the TME to bolster antitumor immunity. Specifically, PPWQ treatment facilitated DC maturation, increased the proportion of memory CTLs, and polarized TAMs toward the immune-supportive M1 phenotype. These results highlight PPWQ’s potential as a multifunctional therapeutic agent, integrating radiotherapy sensitization with metabolic and immune modulation. By addressing the core mechanisms of tumor radioresistance and immune evasion, PPWQ provides a promising avenue for improving RT efficacy and advancing immunoradiotherapy.

## Experimental Method

### Synthesis of a Polyphenol Polymer

The synthesis of the PEG–polyphenol derivative, essential for subsequent metal ion coordination, was adapted and optimized from previously established protocols.^30,31^ Initially, an eight-arm PEG succinimidyl glutarate (tripentaerythritol) (500 mg) was dissolved in 8 mL of anhydrous *N*,*N*-dimethylformamide (DMF) under an inert argon atmosphere to prevent oxidation. Dopamine (190 mg), also dissolved in 2 mL of anhydrous DMF, was added dropwise to the solution while maintaining continuous stirring for 1 h. Triethylamine (95 μL) was subsequently introduced as a catalyst to enhance the efficiency of amide bond formation, and the reaction was allowed to proceed for12 h in a sealed reaction vessel to ensure completion. The following day, a glycine solution (50 mmol L^–1^, pH = 3) was added to quench any unreacted active esters, thereby stabilizing the derivative. To purify the resulting product, dialysis against distilled water was conducted for 48 h to eliminate impurities, followed by lyophilization, yielding the PEG–polyphenol derivative with high purity.

### Preparation of PPWQ NPs

The preparation of PPWQ NPs involved a multistep self-assembly process. First, the PEG–polyphenol derivative (20 μL, 20 mg mL^–1^), ^D^C-^D^PPA peptide, and QR (dissolved in methanol) were mixed in 5 mL of distilled water under gentle stirring for 10 min to promote initial interactions among components. Subsequently, a tungsten hexachloride (WCl_6_) solution, prepared in methanol, was added dropwise over 30 min while maintaining continuous agitation. This step facilitated the metal-phenolic coordination necessary for NP self-assembly. The resulting complex was purified by repeated washing and centrifugation to remove unbound reactants. Additional dialysis for 4 h ensured the elimination of residual solvents and small molecules. The final product, designated as PPWQ, was stored at 4 °C to maintain stability and was characterized for further applications.

### W^6+^ Loading and Release Studies

To determine the efficiency of W^6+^ incorporation with polyphenol, varying amounts of W^6+^ were combined with 30 mg of PP in methanol at weight ratios of PP:WCl_6_:QR = 30:1:1, 30:5:1, and 30:10:1. The mixtures were sonicated for 10 min to ensure thorough dispersion. The metal content was measured using ICP-MS, and the W^6+^ loading efficiency was calculated using the formula: (mass of W^6+^/total mass of PPWQ) × 100%.

### Cytotoxicity and Cell Apoptosis Assay

#### Cytotoxicity Evaluation

The cytotoxic potential of PPWQ NPs was assessed using an MTT assay to determine their effect on 4T1 cells, a widely used breast cancer cell line. Cells were seeded at a density of 5000 cells well^–1^ in 96-well plates and incubated for 12 h to allow adherence. Treatments included various formulations with or without X-ray irradiation (6 Gy), and incubation continued for an additional 24 h. Cell viability was quantified by measuring absorbance at 570 nm, following the standard MTT protocol, providing insights into the dose-dependent cytotoxic effects of PPWQ and its components.

#### Apoptosis Analysis

Apoptosis induction was evaluated using flow cytometry with Annexin V-FITC and propidium iodide (PI) staining. 4T1 cells were seeded in 6-well plates at a density of 2 × 10^5^ cells well^–1^ and exposed to different formulations in fresh RPMI-1640 medium. Following X-ray irradiation (6 Gy) and a 24-h incubation period, the cells were stained to differentiate apoptotic from necrotic cells. Flow cytometry was performed on a Beckman CytoFlex S cytometer, and data analysis was conducted using *FlowJo 10.0* software. This method provided quantitative insights into the apoptotic effects of PPWQ, both as a standalone treatment and in combination with radiotherapy.

### Animal Tumor Model

To establish the orthotopic 4T1 tumor model, 1 × 10^6^ 4T1 cells were implanted into the fourth right mammary fat pad of female Balb/c mice to simulate in situ breast tumor growth. This orthotopic approach allows tumors to develop in their natural microenvironment, enabling more accurate assessment of localized therapies and tumor-immune interactions. All animal procedures were conducted in strict compliance with the Guidelines for the Care and Use of Laboratory Animals at Xiamen University. Ethical approval for the study was obtained from the Xiamen University Experimental Animal Care and Ethics Committee (Approval No. XMULAC20230222). Mice were closely monitored for health status, body weight, and tumor progression to ensure humane treatment and experimental reliability.

### In Vivo Fluorescence Imaging and Biodistribution Analysis

When orthotopic tumors reached an approximate volume of 100 mm^3^, ICG-labeled PPWQ NPs were administered intravenously to evaluate their biodistribution and tumor-targeting efficiency. Fluorescence imaging was conducted using a PerkinElmer imaging system at multiple time points: 2, 4, 8, 12, 24, and 48 h postinjection. This sequential imaging approach allowed for dynamic monitoring of NP accumulation in tumor tissues and clearance from systemic circulation. At 48 h postinjection, tumors and major organs, including the liver, lungs, spleen, and kidneys, were harvested from selected mice for ex vivo fluorescence imaging. The biodistribution analysis provided quantitative insights into the preferential accumulation of PPWQ in tumor tissues compared to nontarget organs, confirming its tumor-targeting specificity and systemic compatibility.

### Bone-Marrow-Derived Dendritic Cell (BMDC) Isolation and Coculture with Cancer Cells

BMDCs were derived from the femurs and tibias of female Balb/c mice aged 8–10 weeks, following a standard protocol. The femurs and tibias were sterilized and washed with RPMI 1640 medium to harvest bone marrow monocytes, and red blood cells were lysed using ACK buffer. The monocytes were maintained in a RPMI 1640 medium containing 10 ng mL^–1^ IL-4 and 20 ng mL^–1^ GM-CSF for 5 days to induce differentiation into BMDCs. To assess the impact of different cancer treatments on DC maturation, treated cancer cells were cocultured with BMDCs at a 1:1 ratio for 24 h. BMDC maturation was evaluated by flow cytometry, gating on the CD11c^+^ population to identify mature DCs (CD11c^+^CD80^+^CD86^+^), which are critical for initiating adaptive immune responses.

### Flow Cytometric Analysis of TME

Orthotopic breast cancer models were established as previously described, and mice were divided into six groups receiving various intravenous treatments. Three days after the final treatment, TDLNs were harvested, dissociated into single-cell suspensions, and stained with antibodies targeting CD11c, CD80, and CD86 to evaluate DC activation.

Tumors were enzymatically digested in RPMI 1640 medium containing 10% FBS, DNase I (Roche), hyaluronidase (Solarbio Life Sciences), collagenase type IV (Gibco), and MgCl_2_·6H_2_O (Aladdin) to obtain single-cell suspensions. These suspensions were filtered and labeled with antibodies targeting CD45, CD11b, F4/80, CD206, CD3, CD4, and CD8, following the manufacturer’s protocol. Flow cytometry was employed to analyze immune cell populations, including T cells, macrophages, and other immune subsets within the TME. This analysis provided detailed insights into immune activation and the polarization of macrophages, which play a pivotal role in the immunosuppressive or immune-supportive state of the TME.

### Statistical Analysis

Statistical analyses were performed using *GraphPad Prism* software. Group differences were assessed using one-way ANOVA, with statistical significance defined at varying levels (*p < 0.05, **p < 0.01, ***p < 0.001, and ****p < 0.0001). Data were presented as mean ± standard deviation (SD) to ensure transparency and reproducibility.

## Data Availability

The authors declare that the data supporting the findings of this study are available in the paper. Any relevant data sets are properly cited in the reference section.

## Supporting Information Available

Image of the full gel and blot of WB data, flow cytometry gating strategies, tumor accumulation behavior of PPWQ NPs, and additional figures and data for the paper (PDF)
