# Supplementary material for: Metal–Phenolic Nanomedicines Targeting Fatty Acid Metabolic Reprogramming to Overcome Immunosuppression in Radiometabolic Cancer Therapy
Source: ACS Appl Mater Interfaces. 2025 Jan 28;17(5):7478–88. doi: 10.1021/acsami.4c21028 (PMC11803545; doi:10.1021/acsami.4c21028)
Supplement: Supplementary file 1 — am4c21028_si_001.pdf [file am4c21028_si_001.pdf]

Supporting Information

Metal-Phenolic Nanomedicines Targeting Fatty Acid Metabolic Reprogramming to Overcome Immunosuppression in Radio-Metabolic Cancer Therapy

Guohao Wang,[‡a] *Dongmei Wang,*[‡b] *Lu Xia,* [‡a] *Jiabian Lian,*[c,d] *Qing Zhang,*[e] *Dongyan Shen,*[a] *Zhanxiang Wang*[*f] *and Yunlu Dai*[*g,h]

a. Xiamen Cell Therapy Research Center, The First Affiliated Hospital of Xiamen University, School of Medicine, Xiamen University, Xiamen, 361003, China.
b. Department of Public Health and Medical Technology, Xiamen Medical College, Xiamen, 361023, China.
c. Center for Precision Medicine, The First Affiliated Hospital, School of Medicine, Xiamen University, Xiamen,361000, China.
d. Department of Laboratory Medicine, The First Affiliated Hospital, School of Medicine, Xiamen University, Xiamen,361000, China.
e. Department of cardiology, the first affiliated hospital of Xiamen university, school of medicine, Xiamen university, Xiamen 361003, China
f. Department of Neurosurgery and Department of Neuroscience, Fujian Key Laboratory of Brain Tumors Diagnosis and Precision Treatment, Xiamen Key Laboratory of Brain Center, the First Affiliated Hospital of Xiamen University, School of Medicine, Xiamen University, Xiamen, 361003, China.
g. Cancer Centre and Institute of Translational Medicine, Faculty of Health Sciences, University of Macau, Macau SAR, 999078, China.
h. MoE Frontiers Science Center for Precision Oncology, University of Macau, Macau SAR, 999078, China.

*To whom correspondence should be addressed. E-mail: yldai@um.edu.mo (Y. Dai); wangzx@xmu.edu.cn (Z. Wang)

‡These authors contributed equally to this work.

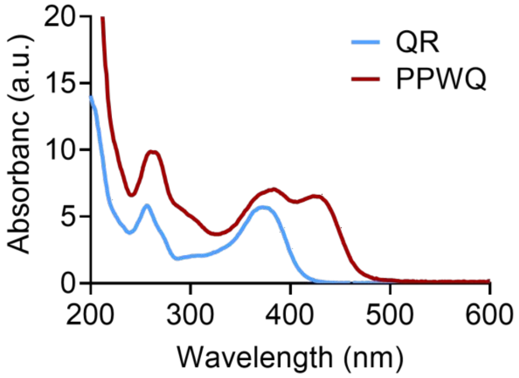

Figure S1. UV-Vis Spectra of QR and PPWQ Nanoparticles.

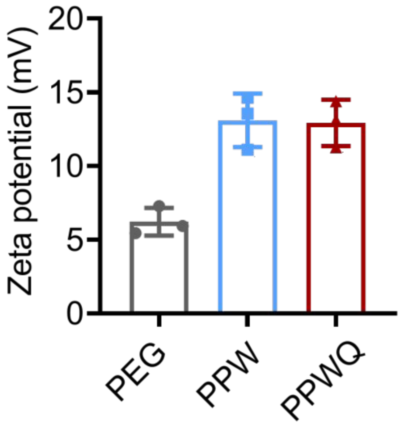

Figure S2. Zeta potential values were recorded for PEG, PPW, and PPWQ nanoparticles to evaluate their surface charge.

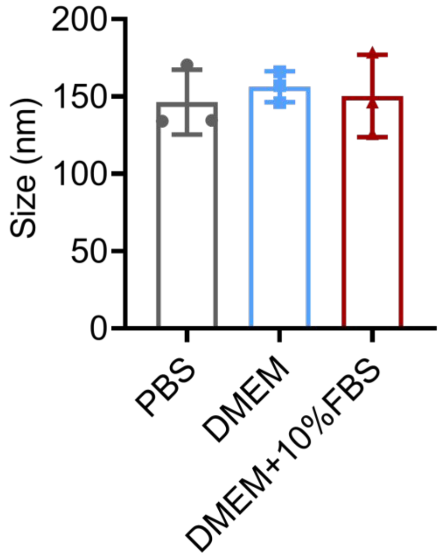

Figure S3. Diameters of PPWQ NPs stored in PBS, DMEM and DMEM containing 10% of fetal bovine serum for 3 days.

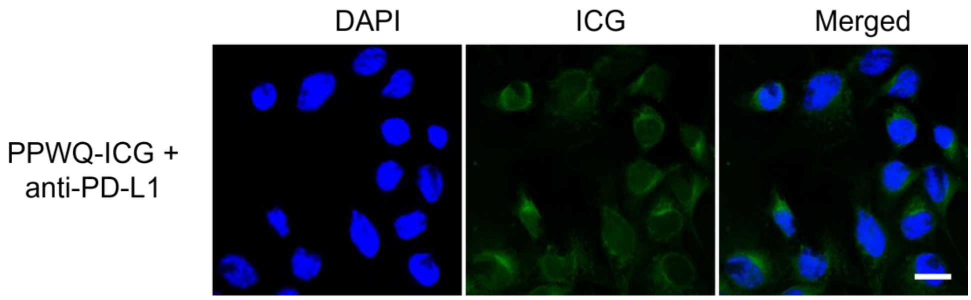

Figure S4. Cell uptake of PPWQ NPs labelling with ICG after anti-PD-L1 blocking. The scale bar denotes 20 μm.

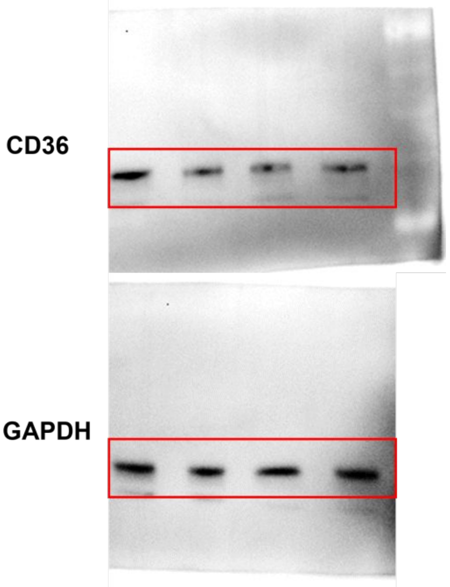

Figure S5. The image of the full gel and blot of Figure. 2b.

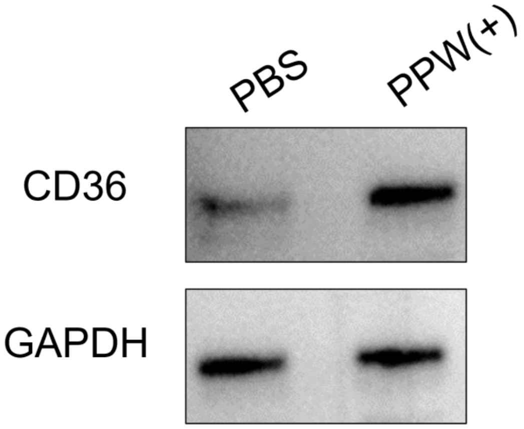

Figure S6. Immunoblot showing CD36 expression in 4T1 cells treated with different agents. GAPDH used as loading control.

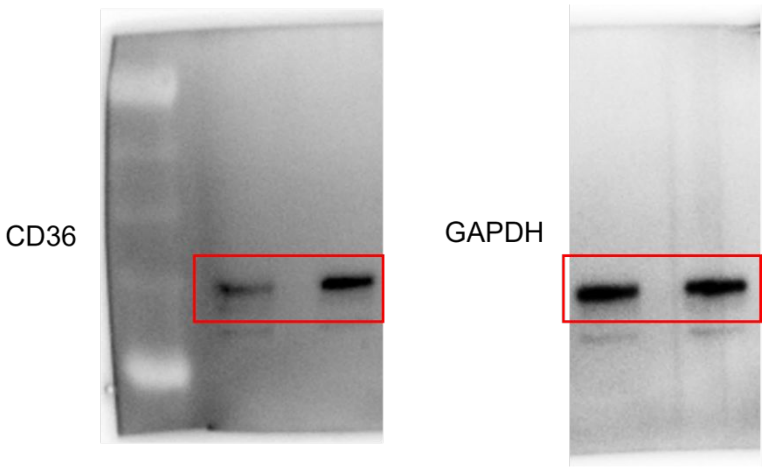

Figure S7. The image of the full gel and blot of Figure S4.

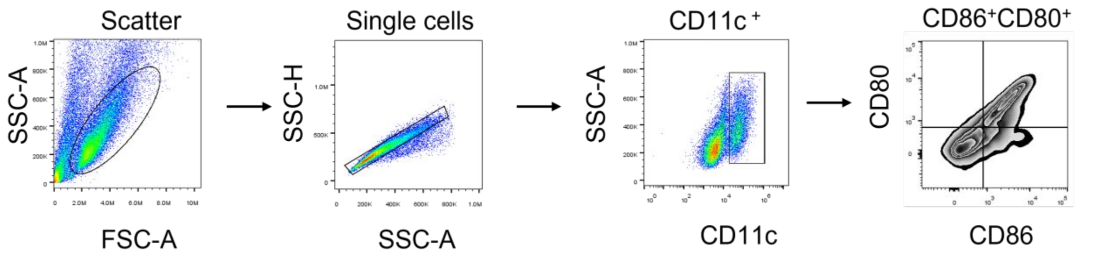

Figure S8. Representative flow cytometry gating strategies for $CD11c^{+}CD80^{+}CD86^{+}$ activated DCs panel.

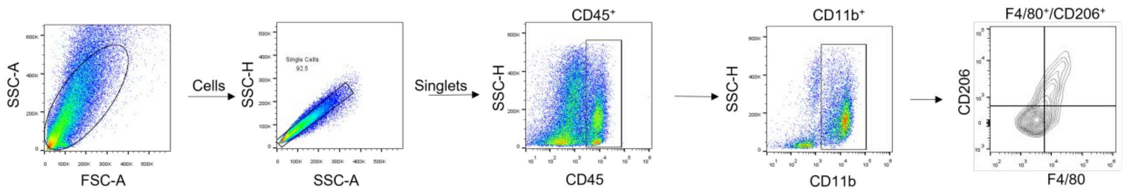

Figure S9. Representative flow cytometry gating strategies for M2-like macrophages panel.

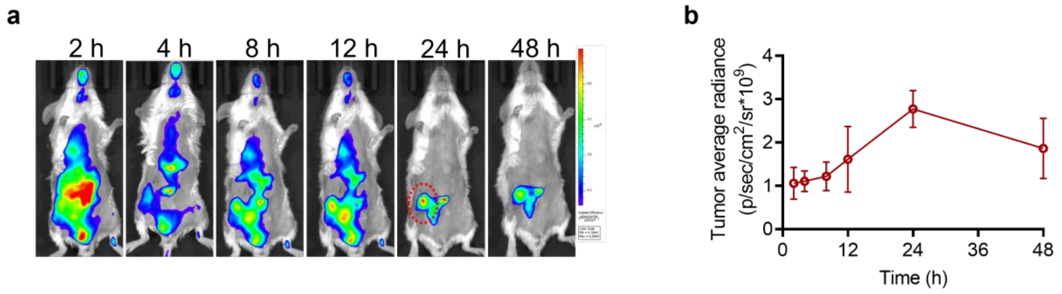

Figure S10. Tumor accumulation behavior of PPWQ NPs. a,b Time-dependent fluorescence images (**a**) and the quantitative analyses on the tumor area (**b**) of 4T1 tumor-bearing mice after intravenous injection of PPWQ NPs (with ICG labelling on PEG) (n =3).

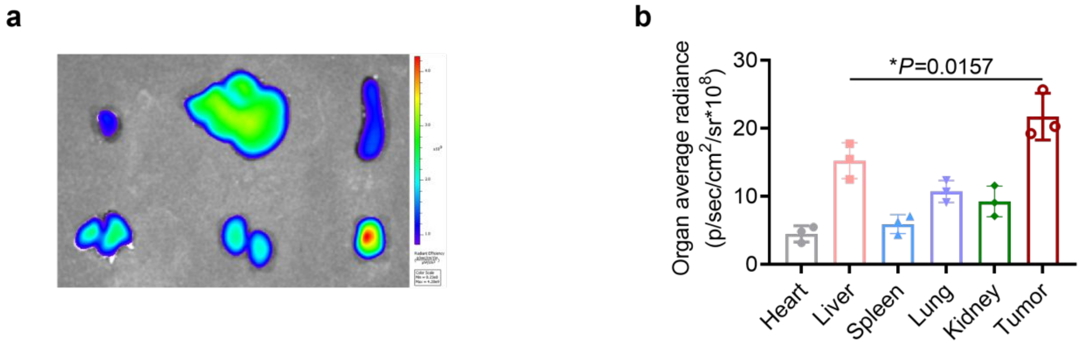

Figure S11. a,b Fluorescence images (**a**) and quantification (**b**) of the corresponding fluorescence intensity of major organs and tumors that were collected from mice receiving PPWQ NPs (with ICG labelling on PEG) at 48 h after administration and were captured using an IVIS Spectrum Imaging System (Perkin Elmer).

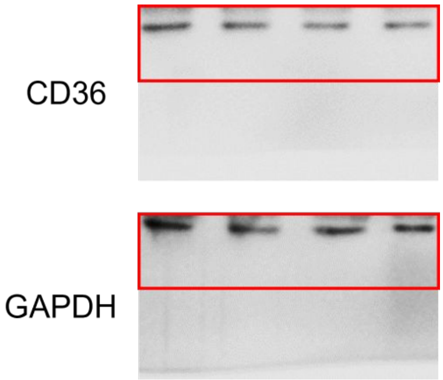

Figure S12. The image of the full gel and blot of Figure. 5b.

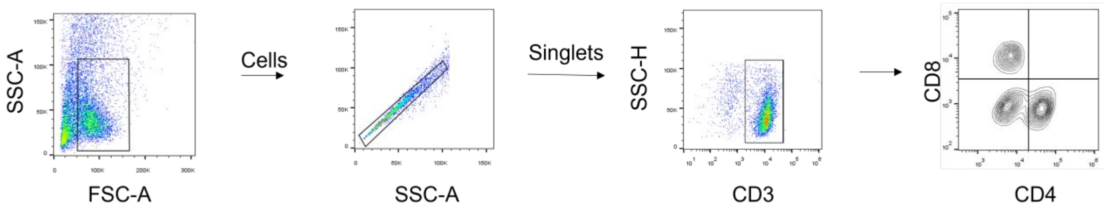

Figure S13. Representative flow cytometry gating strategies for CD8$^+$ T cells and CD4$^+$ T cells T cells in tumor tissue.

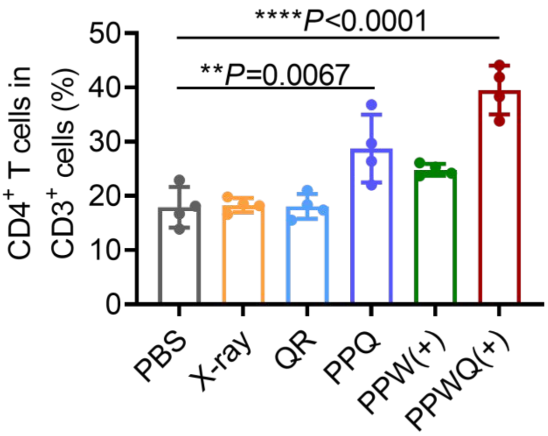

Figure S14. Representative flow cytometry quantification of $CD4^+$ T cells after gating on $CD45^+CD3^+$ cells in tumor tissues.

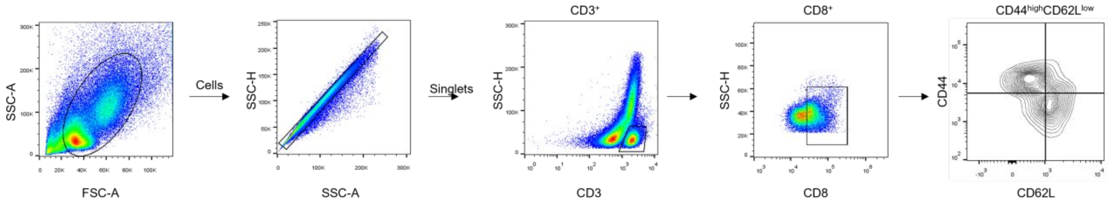

Figure S15. Representative flow cytometry gating strategies for $CD3^+CD8^+CD44^+CD62L^-$ T cells panel in spleen.

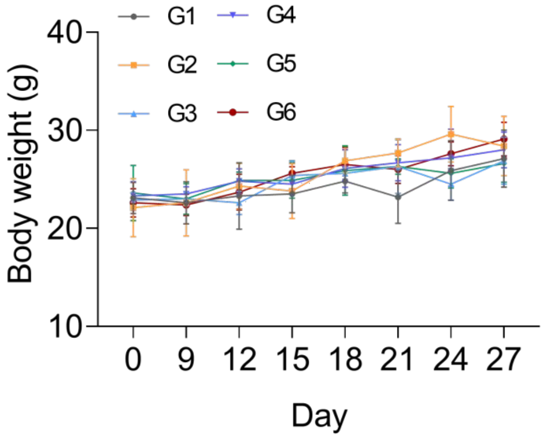

Figure S16. Body weight monitoring of mice during the treatment period.

Table S1. W^{6+} Loading efficiency in PPWQ

PEG-P:WCl_6:QR (w/w)	W^{6+} Loading content (wt %)	Loading efficiency (%)
30:1:1	2.51	80.29
30:5:1	11.99	86.31
30:10:1	16.89	69.24
